# Phenolic Compounds of *Catalpa speciosa*, *Taxus cuspidata*, and *Magnolia acuminata* have Antioxidant and Anticancer Activity

**DOI:** 10.3390/molecules24030412

**Published:** 2019-01-23

**Authors:** Hosam O. Elansary, Agnieszka Szopa, Paweł Kubica, Fahed A. Al-Mana, Eman A. Mahmoud, Tarek K. Ali Zin El-Abedin, Mohamed A. Mattar, Halina Ekiert

**Affiliations:** 1 Plant production Department, College of Food and Agriculture Sciences, King Saud University, Riyadh 11451, Saudi Arabia; falmana@ksu.edu.sa; 2 Floriculture, Ornamental Horticulture and Garden Design, Faculty of Agriculture, Alexandria University, Alexandria 00203, Egypt; 3 Department of Geography, Environmental Management and Energy Studies, University of Johannesburg, Auckland Park Kingsway Campus (APK) Campus, Johannesburg 2006, South Africa; 4 Department of Pharmaceutical Botany, Medical College, Jagiellonian University, ul. Medyczna 9, 30-688 Kraków, Poland; a.szopa@uj.edu.pl (A.S.); p.kubica@uj.edu.pl (P.K.); mfekiert@cyf-kr.edu.pl (H.E.); 5 Department of Food Industries, Faculty of Agriculture, Damietta University, Damietta 34511, Egypt; emanmail2005@yahoo.com; 6 Department of Agricultural Engineering, College of Food and Agriculture Sciences, King Saud University, Riyadh 11451, Saudi Arabia; tkamalzein@ksu.edu.sa (T.K.A.Z.E.-A.); mmattar@ksu.edu.sa (M.A.M.)

**Keywords:** *Catalpa speciosa*, *Taxus cuspidata*, *Magnolia acuminata*, phenols, antioxidants, anticancer

## Abstract

Tree bark represents an important source of medicinal compounds that may be useful for cancer therapy. In the current study, high-performance liquid chromatography with diode-array detection (HPLC-DAD) was used to determine the profile of the phenolic compounds of *Catalpa speciosa**,** Taxus cuspidata*, and *Magnolia acuminata* bark extracts. The antioxidant and anticancer bioactivities against different cancer cell lines were investigated. *M. acuminata* exerted significantly higher antioxidant activities in the diphenyl picrylhydrazine and β-carotene-linoleic acid assays than the other species. In *C. speciosa,* novel profiles of phenolic acids (ferulic acid was the predominant compound) and catechin were detected. In *T. cuspidata*, six phenolic acids were detected; the predominant compounds were hydroxycaffeic acid and protocatechuic acid. In *M. acuminata*, two phenolic acids and three catechins were detected; catechin was the predominant compound. The three species exerted clear anticancer activity against MCF-7, HeLa, Jurkat, T24, and HT-29 cells, with the strongest activity found in the extracts from *M. acuminata*. No antiproliferative activity against normal cells was found. Flow cytometry revealed greater accumulation of necrotic and early/late apoptotic cells in various treated cancer cells than in untreated control cells, and protocatechuic acid induced a similar accumulation of necrotic cells to that of the bark extracts. Caspase-3 and -7 activity was increased in cancer cells treated with different bark extracts; the highest activity was found in the *M. acuminata* treatment. Our results suggested that the treatment of cancer cells with bark extracts of *M. acuminata, C. speciosa,* and *T. cuspidata*, and protocatechuic acid induced apoptosis, suggesting an association between anticancer activities and individual phenolic compounds.

## 1. Introduction

Tree bark is used widely in folklore medicine to treat ailments and control the progress of many diseases and injuries, such as arthritis, gonorrhea, rheumatism, dysentery, malaria, inflammation, wounds, ulcers, and constipation [1,2,3]. Recent investigations found that tree bark may contain antioxidant and anticancer phenolic compounds [2,4,5,6]. However, the current knowledge of the pharmaceutical properties of tree bark is limited.

*Catalpa* belongs to Bignoniaceae and the genus contains several species, including *Catalpa speciosa*, which are distributed throughout North America and some parts of Europe [7]. Leaf extracts from other *Catalpa* species, including *C. ovata, C. fargesii*, *C. bignonioides*, and *C. bungei* are known to have antioxidant activity [8,9] and high phenolic composition. No pharmaceutical studies of *C. speciosa* have been conducted.

*Magnolia* species, which belong to Magnoliaceae, contain several species, including *M. officinalis, M. obovata*, and *M. biondii,* which have been used in folklore medicine for thousands of years in Asia [10]. Moreover, *Magnoliae officinalis cortex* is listed in the newest European Pharmacopoeia 9.0 as an official pharmaceutical raw plant material in Europe [11]. The dried parts of the magnolia are used to control diarrhea, abdominal swelling, or constipation, and cough [12]. The analyses of the bark and/or seeds of *M. officinalis, M. obovata*, and *M. biondii* revealed the availability of specific bioactive compounds, such as magnolol, honokiol, and obovatol, which have potent anticancer and antioxidant activities [4,6]. Other species, such as *M. acuminata*, have attracted little attention.

*Taxus* belongs to Taxaceae and contains approximately 13 species distributed throughout Asia, Europe, and North America [13]. The leaves and bark of some species. such as *Taxus wallichiana*, are used in the traditional Ayurveda and Unani medicines for the control of fever, cough, and cold, and exert anticancer activities [5]. The bark of *Taxus baccata* is a well-known pharmaceutical raw material that contains paclitaxel, a known anticancer compound [11]. *T. cuspidata* bark extract showed anticancer activities due to the presence of paclitaxel and other lignans in the roots [14,15]. However, no studies revealed the detailed phenolic and catechin profiles of this species. 

The current study explores the phenolic, catechin, and flavonoid content of *C. speciosa, T. cuspidata,* and *M. acuminata* using HPLC-DAD method. The antioxidant, antiproliferative, apoptotic, and caspase-3/7 activities have been explored using several cancer cell lines. 

## 2. Results

### 2.1. Targeted Profiling of Catechins and Phenols 

#### 2.1.1. *C. speciosa*

In *C. speciosa* methanolic bark extract, seven phenolic acids (caffeic acid, p-coumaric acid, ferulic acid, gallic acid, p-hydroxybenzoic acid, protocatechuic acid, and vanillic acid) were found out of the 22 screened (Table 1 and Supplementary files). Ferulic acid was the predominant compound (22.7 ± 0.18 mg 100 g^−1^ DW); other phenolic acids were detected in lower quantities. The content of p-hydroxybenzoic acid and vanillic acid was approximately 6 mg 100 g^−1^ DW, the content of caffeic acid, p-coumaric acid, and protocatechuic acid was approximately 3 mg 100 g^−1^ DW, and gallic acid was present at the lowest concentration (ca. 1.6 mg 100 g^−1^ DW). A low amount of catechin (ca. 1.2 mg 100 g^−1^ DW) was detected out of the five analyzed catechin derivatives (Table 2). No flavonoids were detected.

#### 2.1.2. *T. cuspidata*

In *T. cuspidata* bark extracts, caffeic acid, chlorogenic acid, gallic acid, p-hydroxybenzoic acid, hydroxycaffeic acid, and protocatechuic acid were identified (Table 1 and Supplementary files). The predominant compounds were protocatechuic acid (ca. 21 mg 100 g^−1^ DW) and hydroxycaffeic acid (ca. 24 mg 100 g^−1^ DW). In the extracts, no flavonoids or catechins were found. 

#### 2.1.3. *M. acuminata*

In the *M. acuminata* bark extracts, protocatechuic acid (ca. 15 mg 100 g^−1^ DW) was the dominant phenolic acid (Table 1 and Supplementary files). A low composition of ellagic acid (less than 0.5 mg 100 g^−1^ DW) was also identified. However, catechins and catechin derivatives, epicatechin, and epigallocatechin gallate, were found in the extracts (Table 2). Catechin was the main compound (ca. 85.5 mg 100 g^−1^ DW), followed by epicatechin (ca. 23 mg 100 g^−1^ DW) (Table 2). No flavonoids were detected by using the HPLC-DAD method. 

### 2.2. Antioxidant Activities 

Bark extracts showed antioxidant activity as found in Table 3. *M. acuminata* exhibited the highest antioxidant activities in the diphenyl picryl hydrazyl (DPPH) (IC_50_, 3.1 µg mL^−1^) and β-carotene-linoleic acid (IC_50_, 3.6 µg mL^−1^) assays compared to other species. *T. cuspidata* exhibited higher antioxidant activities than *C. speciosa*. *M. acuminata* antioxidant power was comparable with those of the standard antioxidant (BHT). 

### 2.3. Antiproliferative Activities 

The bark extracts exhibited antiproliferative activities against different cancer cell lines, as shown in Table 4. Anticancer activity against MCF-7, HeLa, Jurkat, T24, and HT-29 cells was noted. The strongest anticancer bioactivity was found in *M. acuminata* (IC_50_, 16.20–152.8 µg/mL). Further, no extract exerted antiproliferative activity against normal HEK-293 cells. Specific catechins and phenolics found in the bark extracts, including protocatechuic acid, catechin, ferulic acid, and hydroxycaffeic acid, exhibited notable antiproliferative activity against most cancer cells. 

### 2.4. Apoptotic Cell Population

**Figure 1:**
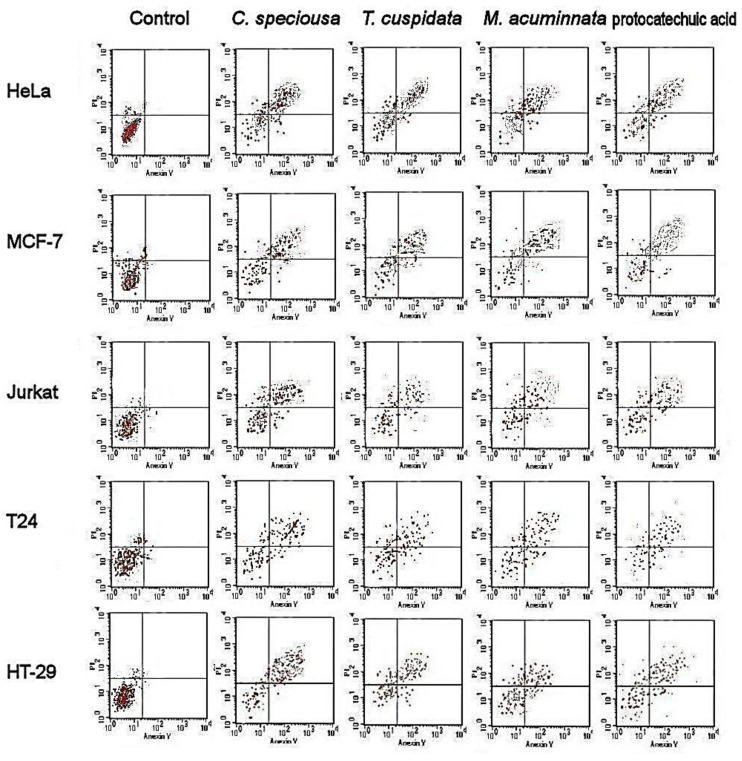
Cellular apoptosis induced in cancer cells at 24 h following treatment with bark extracts and catechin. Lower left, viable cells; upper left, necrotic cells; lower right, early apoptotic cells; and upper right, late apoptotic cells.

**Figure 2:**
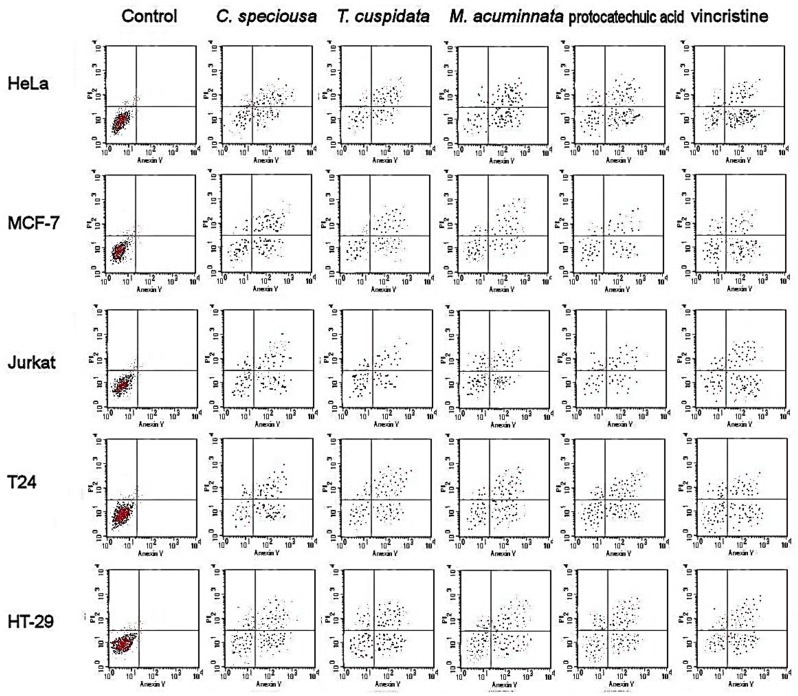
Cellular apoptosis induced in cancer cells at 48 h following treatment with bark extracts and catechin. Lower left, viable cells; upper left, necrotic cells; lower right, early apoptotic cells; and upper right, late apoptotic cells.

Flow cytometry revealed the extent of apoptosis in different cell lines subjected to the (50% Inhibition concentration) IC_50_ of bark extracts (Figure 1 and Figure 2). The results showed a greater accumulation of necrotic cells, and early and late apoptotic cells in treated cancer cells than in control cells in various cancer cell lines. Treatment with protocatechuic acid showed similar accumulation of necrotic cells as treatment with the bark extracts of the three species. The bioactivity of the extracts and protocatechuic acid was sustained after 48 h.

### 2.5. Detection of Caspase-3/7 Activity

**Figure 3:**
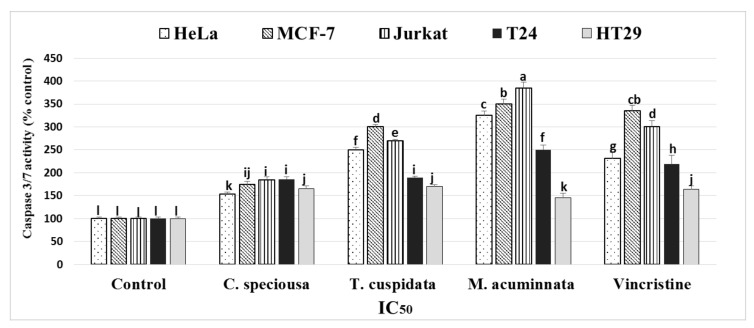
Enzyme activity of caspase 3/7 following treatment of different cancer cells with *C. speciousa*, *T. cuspidate*, and *M. acuminnata* bark extracts (IC_50_). The activity was expressed as a percentage (%) of untreated cells.

The effects of bark extracts on caspase-3/7 activity were investigated in HeLa, MCF-7, Jurkat, HT-29, and T24 cells (Figure 3). The results showed that greater caspase 3/7 activity occurred after *M. acuminata*, *T. cuspidata*, and *C. speciosa* treatment in all cancer cell lines than in the untreated control cells. *M. acuminata* resulted in a higher activity of caspase 3/7 than the other treatments.

### 2.6. Western Blotting Analyses of Caspases-3 and Caspase-7

**Figure 4:**
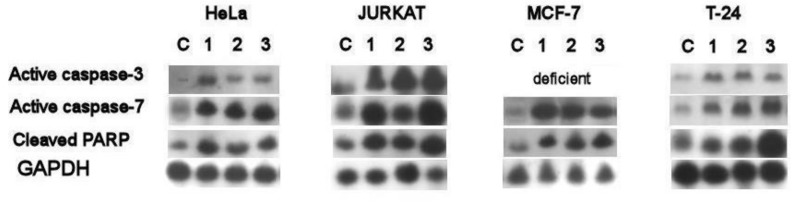
Western blot analysis of active caspase-3, caspase-7 and cleaved PARP using bark extracts (IC_50_) of *C. speciousa* (1), *T. cuspidata* (2), and *M. acuminnata* (3).

Western blotting of caspase-3 and caspase-7 activation by bark extracts was tested as shown in Figure 4. Bark extracts increased the activation of caspase-3 and caspase-7 in all cancer cells, except in MCF-7 which was deficient for caspase-3. Further, high proteolytic cleavage of the nuclear enzyme poly (adenosine diphosphate ribose) polymerase/PARP (caspase substrates) was found in all treatments compared to control. This increased cleavage is required for the activation of key apoptosis executioners (caspase-3 and caspase-7).

## 3. Discussion

This is the first report to document detailed phenolic and catechin profiles of *C. speciosa* bark extract. The phenolic acid profile of *C. speciosa* bark was relatively unique (compared to other species) and contained ferulic acid (as the dominant compound), caffeic acid, p-coumaric acid, gallic acid, p-hydroxybenzoic acid, protocatechuic acid, and vanillic acid. Previous investigations into *C. ovata, C. fargesii*, and *C. bungei* leaves [8] have revealed only flavonoids such as luteolin and apigenin. *C. bignonioides* flowers and leaves had high phenolic contents [9]. The current investigation was the first to identify several phenolics in the *T. cuspidata* bark extract. Previously detected paclitaxel, a diterpenoid with anticancer activity, in the *T. cuspidata* bark extract [14]. In addition, several lignans were isolated from other parts of this species [15]. In different species (*Taxus wallichiana* Zucc.), the leaves and bark were found to contain paclitaxel and exhibit potent anticancer activities [5].

In *M. acuminata* bark, two phenolic acids (ellagic acid and protocatechuic acid) and three catechins (catechin, epicatechin, and epigallocatechin gallate) were found. Catechin was the predominant compound, followed by epicatechin; neither had been previously isolated from *M. acuminata.* Previous investigations described other phenols in *M. officinalis* stem bark; these phenols included magnolianone and its derivatives, which exerted antioxidant activities [4,16], and magnoloside, a phenylethanoid glycoside [6].

The antioxidant activity assays showed that *M. acuminata* bark extract had the highest antioxidant activity. This high antioxidant activity was mainly attributable to the high concentration of catechin (85.47 ± 1.30 mg 100 g^−1^) and catechin derivatives in the bark extracts. Catechin is strongly associated with the overall antioxidant activities in various species [17,18]. The barks of other species, such as *Magnolia officinalis*, were shown to have antioxidant activities [19], which were attributed to the glycosides and flavonoids in another study [20]. Studied on other species, such as *Magnolia grandiflora*, it revealed antioxidant effect in the flower extract [21] and comparable activity in the leaf extract [22]. *T. cuspidata* exerted stronger antioxidant activities than *C. speciosa*. A study on *T. cuspidata* bark revealed polyphenols that might have antioxidant effects, including lignans and catechins [23]; the authors found catechin and epicatechin, although we did not detect these types of catechins in the current study. In *C. speciosa*, we found a moderate antioxidant effect for the bark. Previous investigations revealed the antioxidant effect of the leaves of *C. ovata, C. fargesii*, and *C. bungei* [8], as well as in the inflorescence and leaves of *C. bignonioides* [9]. These antioxidant activities were attributed to the high total phenolic content [9] or the presence of flavonoids such as luteolin and apigenin [8]. In our study, this antioxidant bioactivity of *C. speciosa* bark extract was attributed to specific phenolic contents, including ferulic acid (22.7 ± 0.18 mg 100 g^−1^), which are known to be strong antioxidants [24].

Anticancer activities against the MCF-7, HeLa, Jurkat, T24, and HT-29 cells were found; the highest activities were exhibited by *M. acuminata.* The main components detected in *M. acuminata* were catechins, which were relatively abundant, including catechin, epicatechin, protocatechuic acid, and epigallocatechin gallate; the observed effects were related to these components of the bark. Protocatechuic acid (PCA, 3,4-dihydroxybenzoic acid) has been correlated to anticancer activities against different cancer cells, as found in this investigation, and is a relatively strong antioxidant [25,26]. Catechins are strongly associated with anticancer effects, such as reports of green tea catechins [27] and the synergistic effects with other treatments [28]. Epicatechin is also associated with the anticancer activity and the induction of apoptosis [29], especially epicatechin oligomers [30]. In addition, it is believed that epicatechin and epigallocatechin in green tea have anticancer and apoptosis-inducing activities [31]. Other species of *Magnolia* also have anticancer activities [32].

The anticancer bioactivity of *C. speciosa* is correlated with its major phenolic compartments, including ferulic acid, p-hydroxybenzoic acid, vanillic acid, and caffeic acid. Ferulic acid suppresses the metastasis in breast cancer cells by regulating the reversal of epithelial-mesenchymal transition [33] and inducing the cell cycle arrest in the cells of cervical cancer [34]. In our study, ferulic acid was the major phenol found in the bark extract (22.7 ± 0.18 mg 100 g^−1^) and is greatly associated with anticancer activity. We found that *C. speciosa* bark extracts have potential antioxidant and anticancer activities against MCF-7 breast cancer, which may be attributable to phenolic acid compounds such as protocatechuic acid and p-hydroxybenzoic acid. These compounds were found to have the power to control MCF-7 breast and PC-3 prostate cancers [35].

*T. cuspidata* showed antiproliferative activities against Jurkat, MCF-7, and HeLa cancer cells and these activities were directly associated with the major phenolic and catechin components, including hydroxycaffeic acid (23.98 ± 1.3 mg 100 g^−1^), chlorogenic acid (8.3 ± 0.22 mg 100 g^−1^), protocatechuic acid (20.97 ± 0.56 mg 100 g^−1^), and gallic acid (2.04 ± 0.07 mg 100 g^−1^). These phenolic and catechin compounds were shown to have antiproliferative effects against different cancer cells [35,36]. In addition to our results that the bark extracts had clear antiproliferative activities against specific cancer cell types, it was previously reported that twigs and needles of the same plant exert anticancer activities against human cancer [37].

The results of flow cytometry showed an accumulation of necrotic cells and early and late apoptotic cells in various cancer cells subjected to bark extracts compared with the untreated control cells. The apoptotic activities of *M. acuminata* are strongly connected with the catechin composition, as similar effects to those of catechin were found. This conclusion was strongly supported by the apoptosis induction described before for catechins such as epicatechin [29], epicatechin oligomers [30], epicatechin, and epigallocatechin [31]. The flower extract of *M. grandiflora* showed an apoptotic effect in lymphocytic leukemia cells [38]. There was a high accumulation of early and late apoptotic cells after following bark extracts and catechin treatment. In *C. speciosa*, ferulic acid was suggested to be the cornerstone of the apoptosis-inducing activities as it was associated with the death of osteosarcoma cells through the promotion of caspase-3 and apoptosis [39]. In another study, ferulic acid induced cell cycle arrest in cervical cancer cells [34]. In *T. cuspidata*, the apoptotic activities observed in this study agreed with a previous investigation into the needle and twig extracts of the same species [37]. The authors reported HeLa cells apoptosis in HeLa cells and low toxicity in normal cells and G(2)/M cell cycle arrest. The major phenolic acid in *T. cuspidata* is protocatechuic acid which showed apoptosis and slow metastasis in cancer cells [40].

Caspase-3 and -7 enzymes function as mediators of apoptosis through DNA fragmentation and apoptotic chromatin condensation, which lead to cell death [41,42]. We found increased activities of these enzymes in bark extract-treated cancer cells except in MCF-7 which was deficient for caspase-3 only [43,44]. The western plotting of these enzymes confirmed the antiproliferative and apoptotic activities of the three bark extracts as found in in the extracts of other plants [45]. Previous investigations into the use of ferulic acid to control cancer cells revealed that this compound promoted the apoptosis pathway through the activation of caspase-3 [39]. This was the case for *C. speciosa*, in which ferulic acid was the major phenol found in the bark extracts studied. *C. speciosa* might be a potential new natural source of ferulic acid. The highest activities of caspase-3/7 were found after *M. acuminata* treatment and this activity may be associated with the important catechins in this bark, such as epigallocatechin gallate and epicatechin, and the phenolic acid, protocatechuic acid. Epigallocatechin gallate was one of the major catechins found in *M. acuminata* bark in this study and was strongly related to the increased activities of caspase-3 in green tea catechins [46]. Protocatechuic acid was shown to increase caspase-3 activities [40]; however, another study reported contrasting results [25]. Epicatechin is strongly related to caspase-3 activity in several studies [47,48]. *T. cuspidata* showed some degree of caspase-3/7 activation, which may be attributable to several phenolic compounds found in the bark extract that stimulate the activity of caspase-3, such as chlorogenic acid [49] and gallic acid [50].

## 4. Materials and Methods

### 4.1. Plant Material

*Catalpa speciosa* (Bignoniaceae)*, Taxus cuspidata* Siebold & Zucc.(Taxaceae), and *Magnolia acuminata* L. (Magnoliaceae) outer bark was obtained from identified plants at the Arboretum of University of Guelph, Ontario, Canada. The samples were identified by Hosam Elansary and a voucher was deposited at the University of Guelph and Alexandria University (Hosam000980-2018).

### 4.2. Sample Preparation and Cell Cultures

Fresh bark (0.25 g) was dried at 35 °C until a constant weight was obtained. The samples were ground and then dissolved in 3 mL methanol (99%) for 1 h in the dark at 25 °C. Bark solutions were centrifuged for 5 min at 10,000 rpm (7000 × *g*) and the supernatant was obtained (~2.7 mL). The samples were passed through a 0.45 μm polytetrafluroethylene (PTFE) nylon filter and then stored at −80 °C. Analytical grade chemicals (Sigma Aldrich, Germany) were used in the bioassays. The cancer cell lines, including cervical adenocarcinoma (HeLa), breast adenocarcinoma (MCF-7), T-cell lymphoblast like (Jurkat), urinary bladder carcinoma (T24), and colon adenocarcinoma (HT-29), were obtained from the American Type Culture Collection (ATCC).

### 4.3. Analyses of Phenolic Compounds

*C. speciosa*, *T. cuspidata*, and *M. acuminata* bark samples were dried by lyophilization (Labconco, Kansas City, MO, USA) and powdered. Bark samples were extracted (0.5 g) as described before [51]. Validated chromatographic analyses were performed using the HPLC [52,53]. A Purospher^®^ RP-18e analytical column (4 × 250 mm, 5 mL; Merck, Berlin, Germany) was used in the HPLC-DAD (Merck-Hitachi, Tokyo, Japan) equipment. A gradient program was used with a flow rate of 1 mL/min, a detection wavelength of 254 nm, and an injection volume of 10 µL [51,54,55]. UV-DAD spectra and t_r_ values were used for the quantification of the compounds alongside the phenolic, catechin, and flavonoid standards. These included benzoic acid and related derivatives: Ellagic, gallic, 3,4-dihydroxyphenylacetic, protocatechuic, gentisic, p-hydroxybenzoic, salicylic, vanillic, and syringic acids. In addition to cinnamic acid and the related derivatives, such as caffeic, coumaric, ferulic, *o*-coumaric, *m*-coumaric, *p*-hydrocaffeic, isoferulic, sinapic acids, and depsides (chlorogenic, rosmarinic, and neochlorogenic acids). The catechins included catechin, epicatechin, epigallocatechin gallate, epicatechin gallate, and epigallocatechin. The flavonoid standards included aglycones (kaempferol, myricetin, quercetin luteolin, and rhamnetin) and glycosides (apigetrin, cynaroside, robinin, hyperoside, isoquercetin, quercitrin, rutin, trifolin, and vitexin). The standards were obtained from Sigma-Aldrich (Berlin, Germany).

### 4.4. Antioxidant Activity

The DPPH and β-carotene-linoleic acid assays were used in the Faculty of Food and Agricultural Sciences, King Saud University to determine the antioxidant activities of bark extract [56]. For the DPPH assay, the samples were incubated for 30 min and the absorbance of the samples at 517 nm was measured. In the β-carotene-linoleic acid assay, the absorbance of the samples at 470 nm was measured. The concentration of the sample required to scavenge 50% of the DPPH/β-carotene-linoleic acid solutions, the IC_50_ (µg/mL) was determined by plotting the inhibition percentage against extract concentration. A standard antioxidant (butylated hydroxytoluene, BHT) was used as a positive control and the inhibition by the concentration of each sample was compared with that of the BHT and blank. The antioxidant activities were repeated twice in duplicates.

### 4.5. Antiproliferative Activity

The antiproliferative activity of bark extracts was examined in different cancer cells lines (HeLa, MCF-7, Jurkat, HT-29, and T24) and in normal cells (HEK-293) by using a modified 3-(4,5-dimethylthiazol-2-yl)-2,5-diphenyltetrazolium bromide (MTT) method [57]. The cells were grown in 75 cm^2^ flasks in MEM supplemented with 10% FBS, 17.8 mM NaHCO_3_, 0.1 mM non-essential amino acids, and 1 mM sodium pyruvate. They were seeded into 96-well plates at a density of 4  ×  10^−4^ per well, left to stand in 270 µL of medium, and incubated in an atmosphere of 37 °C and 5% CO_2_. Sterilin-filtered leaf extracts were added to the culture media in microtiter plates. Five doses of bark extracts were used at final concentrations of 50, 100, 200, 300, and 400 µg/mL in culture medium. Untreated cells were considered used as negative controls and vinblastine sulfate and taxol treatment were used as the positive controls. After incubation of the culture medium for 2 days at 37 °C and 5% CO_2_, phosphate buffer saline (PBS) washes were performed to remove extract traces and the medium supplied was 12 mM MTT dissolved in PBS. Subsequently, 0.04 N HCl dissolved in isopropanol was mixed in each well, left to stand for 40 min, and the absorbance at 570 nm was determined by using a microplate reader (Thermo Fisher Scientific, Waltham, Massachusetts, USA). The percentage inhibition of antiproliferation activity was calculated in triplicate [58]:

% Inhibition = (Abs.570 nm control—Abs.570 nm sample)/Abs.570 nm control × 100. Subsequently, IC_50_ values were obtained by plotting the percentage of cell viability against the extract concentration and expressed in µg/mL.

### 4.6. Apoptotic Cell Population

Flow cytometry (FAC Scan, Becton Dickinson, Iowa, USA) was used to measure the apoptotic cell population [59]. Different types of cancer cells were cultured (37 °C, 5% CO_2_) in 6-well plates and treated for 24/48 h with the IC_50_ of bark extract, as determined from the MTT assay, and catechin and untreated samples were considered as the control. The cells were detached by using trypsin (0.25%) in Hank’s balanced salt solution (Thermo Fisher Scientific, Berlin, Germany). For staining the cells, the Annexin V apoptosis detection kit (Sigma, St. Louis, MO, USA) was used. Briefly, the cells were incubated in the dark at 37 °C for 15 min and washed with cold PBS, and the apoptotic populations were shown by the flow cytometer in quadrants: Lower left (viable cells), upper left (necrotic cells), lower right (early apoptotic cells), and upper right (late apoptotic cells).

### 4.7. Caspase-Glo 3/7 Assay

The effect of different bark extracts on caspase-3/7 activity using different cancer cell lines was detected by the Caspase-Glo 3/7 Assay kit (Promega, Berlin, Germany). The cancer cell lines were cultured in Roswell Park Memorial Institute (RPMI) growth medium (Sigma-Aldrich, St. Louis, MO, USA) in 96-well plates in the presence of the IC_50_ of the extracts, catechin, or DMSO (solvent control) for 24 h. Caspase-Glo 3/7 reagent (100 µL) was added to each well, mixed, and then incubated at room temperature for 1 h. The luminescence was of each well was detected by using an Infinite M2000 Pro™ (Tecan). The activity of caspase-3/7 was expressed as a percentage (%) of the untreated samples.

### 4.8. Western Blotting of Caspase-3 and Caspase-7

Cancer cells were treated with bark extracts (IC_50_) for 24 h then harvested, washed with PBS, lysed in protease-inhibitor cocktail buffer (Roche Diagnostic, Bern, Switzerland). The supernatant was collected by centrifugation (2500 ×g for 15 min), then the protein was extracted and the concentration was determined by the bicinchoninic acid protein assay kit (Sigma-Aldrich, Berlin, Germany). Sample proteins (60–80) µg were separated by SDS-PAGE (10%) then blotted on a Polyvinylidene difluoride (PVDF) membrane. Blocking of proteins was achieved by treating the membrane with 5% skimmed milk + 1XTBS + 0.1% Tween 20 for 60 min. The membrane was treated with caspase-3 (#9661), Caspase-7 primary antibodies (#9492), poly (adenosine diphosphate ribose) polymerase (PARP) (#9542), and GAPDH (internal control, #sc-32233) (1:1,000; Cell Signaling Technology, Danvers, MA, USA) at 4 °C overnight. The membrane was washed then incubated with anti-rabbit (cat. no. sc-2030)/ anti-mouse (cat. no. sc-2005) secondary antibody (1:2,000; Santa Cruz Biotechnology, Inc. Dallas, Texas, USA). The bands were detected by ECL reagent and GE Healthcare Bio-Sciences AB Image Quant LAS 4000 (GE Healthcare, Berlin, Germany). The results shown are representative of three independent experiments.

### 4.9. Statistical Analyses

The least significant difference (LSD) was computed by using SPSS software (version 22.0, IBM, New York, USA). The quantitative results of the chromatographic analyses are expressed in (mg 100 g^−1^ DW) as the mean ± SD of three series of experiments.

## 5. Conclusions

*M. acuminata* showed significantly higher antioxidant activities than the other species tested and standard antioxidants. In *C. speciosa,* seven phenolic acids (ferulic acid, caffeic acid, p-hydroxybenzoic acid, p-coumaric acid, gallic acid, protocatechuic acid, and vanillic acid) and catechin were detected by using HPLC-DAD analysis. In *T. cuspidata*, five phenolics were detected and the dominant compound was hydroxycaffeic acid. In *M. acuminata*, two phenolic acids (ellagic acid and protocatechuic acid) and three catechins (catechin, epicatechin, and epigallocatechin gallate) were detected; catechin was the predominant compound. The extracts exerted clear anticancer activities against MCF-7, HeLa, Jurkat, T24, and HT-29 cells. The strongest anticancer activity was exerted by the extract of *M. acuminata*. Further, no antiproliferative activities in normal cells were observed. Flow cytometry showed a greater accumulation of necrotic cells and early and late apoptotic cells in various cancer cell lines treated with the extracts compared with the untreated control cells. Protocatechuic acid resulted in similar accumulation of necrotic cells as the bark extracts of the three species. Increased caspase-3/7 activities were observed in cancer cells treated with different bark extracts and the highest activity was induced by *M. acuminata* treatment. In conclusion, our results indicate the induction of apoptosis after the treatment of cancer cells with bark extracts of *M. acuminata, C. speciosa, T. cuspidata*, and protocatechuic acid, and suggest the association between anticancer activities and the individual phenolic constituents.

## Figures and Tables

**Table 1:** The phenolic acid compositions of *Catalpa speciosa*, *Taxus cuspidata*, and *Magnolia acuminnata* outer bark extracts.

Species	Chemical Compound	Amount [mg 100g^−1^] D.W.
*Catalpa speciosa*	Caffeic acid	3.04 ± 0.45
	p-Coumaric acid	3.28 ± 0.44
	Ferulic acid	22.7 ± 0.18
	Gallic acid	1.57 ± 0.04
	p-Hydroxybenzoic acid	6.42 ± 0.03
	Protocatechuic acid	3.22 ± 0.02
	Vanillic acid	5.77 ± 0.22
*Taxus cuspidata*	Caffeic acid	3.05 ± 0.01
	Chlorogenic acid	8.30 ± 0.22
	Gallic acid	2.04 ± 0.07
	p-Hydroxybenzoic acid	2.42 ± 0.16
	Hydroxycaffeic acid	23.98 ± 1.3
	Protocatechuic acid	20.97 ± 0.56
*Magnolia acuminnata*	Ellagic acid	0.43 ± 0.08
	Protocatechuic acid	15.31 ± 1.19

**Table 2:** The catechin derivatives compositions of *Catalpa speciosa* and *Magnolia acuminnata* outer bark extracts.

Species	Chemical Compound	Amount [mg 100g^−1^] D.W.
*Catalpa speciosa*	Catechin	1.19 ± 0.05
*Magnolia acuminnata*	Catechin	85.47 ± 1.30
	Epicatechin	22.78 ± 0.53
	Epigallocatechin gallate	14.22 ± 0.95

**Table 3:** Diphenyl picryl hydrazyl (DPPH) and *β*-carotene-linoleic acid of *Catalpa speciosa*, *Taxus cuspidata*, *Magnolia acuminnata* outer bark extracts.

Plant/Standard	DPPH Free Radical Scavenging Activity (IC50, µg mL^−1^)	β-Carotene-Linoleic Acid Assay (IC50, µg mL^−1^)
***Catalpa speciosa***	4.4 ± 0.1a	5.1 ± 0.1a
***Taxus cuspidata***	4.2 ± 0.1b	4.8 ± 0.1b
***Magnolia acuminnata***	3.1 ± 0.1c	3.6 ± 0.1c
**BHT**	2.9 ± 0.1c	3.2 ± 0.1c

**Table 4:** In vitro antiproliferative activity (IC_50_ (µg/mL)) of *Catalpa speciosa*, *Taxus cuspidata*, *Magnolia acuminnata* outer bark extracts on cancer cell lines.

Plant/Standard	HeLa	MCF-7	Jurkat	T24	HT-29	HEK-293
***Catalpa speciosa***	58.3 ± 1.7	41.19 ± 1.6	41.4 ± 1.1	249.5 ± 2.9	111.5 ± 2.9	˃400
***Taxus cuspidata***	54.5 ± 1.9	39.51 ± 0.9	37.3 ± 0.5	220.1 ± 2.9	102.2 ± 3.1	˃400
***Magnolia acuminnata***	28.4 ± 1.3	16.20 ± 1.1	25.1 ± 1.1	152.8 ± 2.9	89.2 ± 2.5	˃400
**Catechin**	36.48 ± 1.2	17.64 ± 1.8	38.16 ± 0.7	183.28 ± 4.3	96.16 ± 1.5	˃400
**Protocatechuic acid**	39.10 ± 2.3	18.97 ± 2.1	47.35 ± 2.1	176.35 ± 2.1	95.35 ± 3.3	˃400
**Ferulic acid**	51.73 ± 3.5	43.85 ± 1.3	39.11 ± 2.3	227.26 ± 5.1	126.26 ± 4.4	˃400
**Hydroxycaffeic acid**	65.31 ± 2.4	58.11 ± 0.9	63.09 ± 1.4	176.12 ± 3.6	113.15 ± 2.9	˃400
**Vinblastine sulfate**	2.7 ± 0.06	‒	0.1 ± 0.09	65.7 ± 2.1	21.0 ± 0.1	50.1± 2.3
**Vincristine**	8.5 ± 0.1	4.63 ± 1.8	0.4 ± 0.05	89.8 ± 2.5	47.3 ± 0.2	78.3 ± 1.6
**Taxol**	‒	0.09 ± 0.009	‒	‒	‒	‒

## Supplementary Materials

The following are available online at http://www.mdpi.com/1420-3049/24/3/412/s1, Figure S1: The representative HPLC-UV chromatogram (λ = 254 nm) of *Magnolia acuminnata* bark extract; 1 - protocatechuic acid (tR = 6.3 min), 2 – catechin (tR = 8.8 min), 3 - elgaic acid (tR = 12.2 min), 4 - epigallocatechin gallate (tR = 14.8 min), 5 – epicatechin (tR = 19.2 min), Figure S2: The representative HPLC-UV chromatogram (λ = 254 nm) of Taxus cuspidate bark extract; 1 - gallic acid (tR = 3.2 min), 2 - protocatechuic acid (tR = 6.3 min), 3 - chlorogenic acid (tR=9.2 min), 4 - p-hydroxybensoic acid (tR = 11.8 min), 5 - caffeic acid (tR = 15.2 min), 6 - hydroxycaffeic acid (tR = 15.8 min), Figure S3: The representative HPLC-UV chromatogram (λ = 254 nm) of Catalpa speciosa bark extract; 1 - gallic acid (tR = 3.2 min), 2 - protocatechuic acid (tR = 6.3 min), 3 – catechin (tR = 8.8 min), 4- p-hydroxybensoic acid (tR = 11.8 min), 5 - vanillic acid (tR = 12 min), 6 -caffeic acid (tR = 15.2 min), 7 - p-coumaric acid(tR = 26 min), 8 - ferulic acid(tR = 34.4 min).
