# Supplementary material for: Phenolic Compounds of Catalpa speciosa, Taxus cuspidata, and Magnolia acuminata have Antioxidant and Anticancer Activity
Source: Molecules. 2019 Jan 23;24(3):412. doi: 10.3390/molecules24030412 (PMC6384650; doi:10.3390/molecules24030412)

Phenolic Compounds of *Catalpa Speciosa*, *Taxus Cuspidate*, and *Magnolia Acuminata* have Antioxidant and Anticancer Activity

Hosam O. Elansary [1,2,3,*], Agnieszka Szopa [4], Paweł Kubica [4], Fahed A. Al-Mana [1], Eman A. Mahmoud [5], Tarek K. Ali Zin El-Abedin [6], Mohamed A. Mattar [6] and Halina Ekiert [4]

1 Plant production Department, College of Food and Agriculture Sciences, King Saud University, Riyadh 11451, Saudi Arabia; helansary@ksu.edu.sa (H.O.E.); falmana@ksu.edu.sa (F.A.A.M.)
2 Floriculture, Ornamental Horticulture and Garden Design, Faculty of Agriculture, Alexandria University, 00203, Egypt
3 Department of Geography, Environmental Management and Energy Studies, University of Johannesburg, Auckland Park Kingsway Campus (APK) campus, 2006, South Africa
4 Department of Pharmaceutical Botany, Medical College, Jagiellonian University, ul. Medyczna 9, 30-688 Kraków, Poland; a.szopa@uj.edu.pl (A.S.); p.kubica@uj.edu.pl (P.K.); Halina Ekiert (H.E.)
5 Department of Food Industries, Faculty of Agriculture, Damietta University, Damietta 34511, Egypt; emanmail2005@yahoo.com
6 Department of Agricultural Engineering, College of Food and Agriculture Sciences, King Saud University, Riyadh *11451*, Saudi Arabia; tkamalzein@ksu.edu.sa
* Correspondence: helansary@ksu.edu.sa; Tel.: +966 571586389

Received: 06 January 2019; Accepted: 22 January 2019; Published: date

Supplementary Materials:

Supp. Fig.1 The representative HPLC-UV chromatogram (λ = 254 nm) of *Magnolia acuminnata* bark extract; 1 - protocatechuic acid (tR=6.3 min), 2 – catechin (tR=8.8 min), 3 - elgaic acid (tR=12.2 min), 4 - epigallocatechin gallate (tR=14.8 min), 5 – epicatechin (tR=19.2 min).

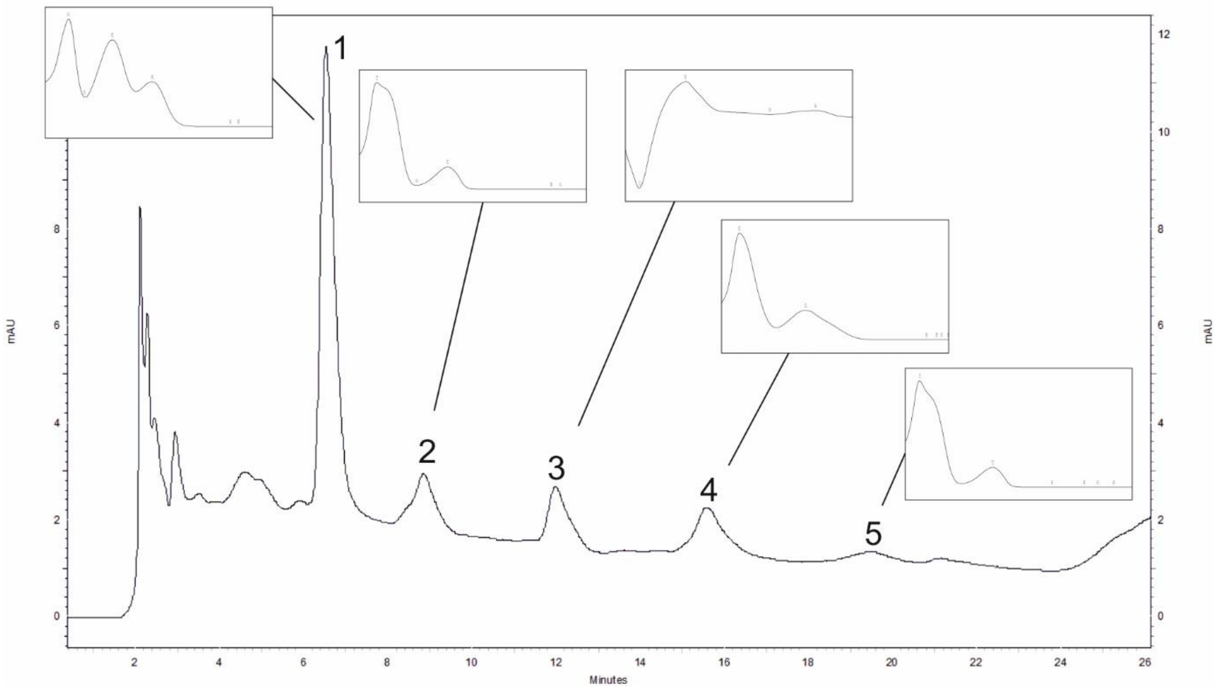

Supp. Fig.2 The representative HPLC-UV chromatogram (λ = 254 nm) of *Taxus cuspidate* bark extract; 1 - gallic acid (tR=3.2 min), 2 - protocatechuic acid (tR=6.3 min), 3 - chlorogenic acid (tR=9.2 min), 4 - p-hydroxybensoic acid (tR=11.8 min), 5 - caffeic acid (tR=15.2 min), 6 - hydroxycaffeic acid (tR=15.8 min).

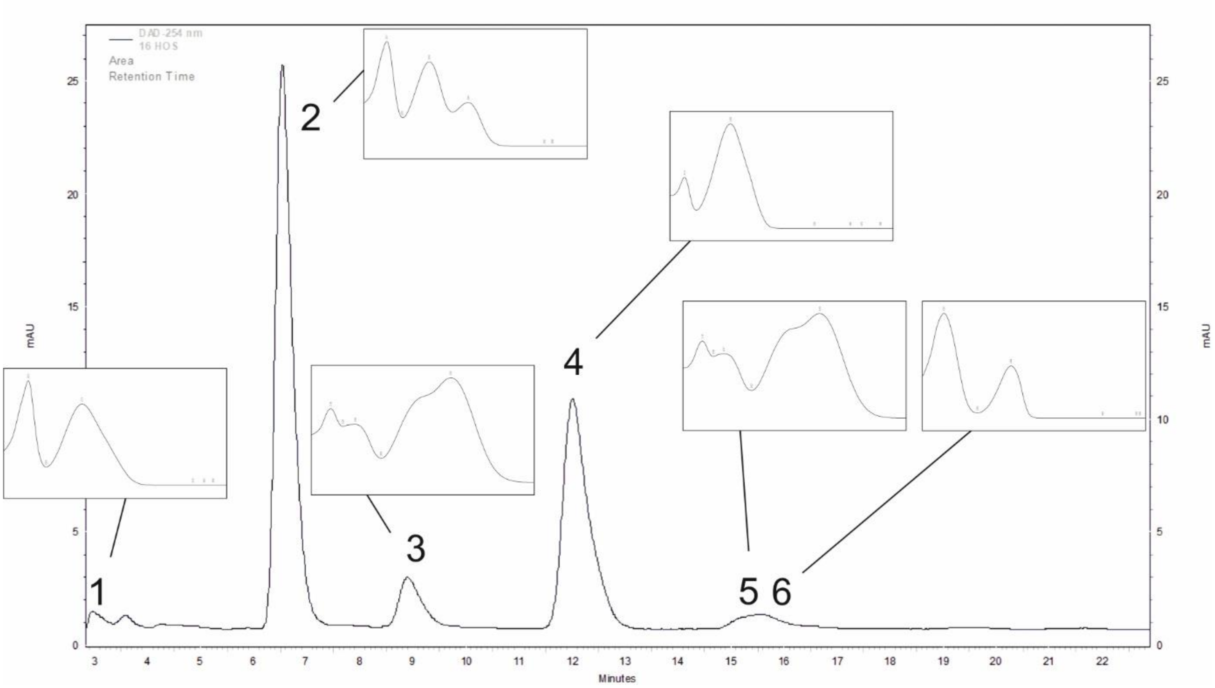

Supp. Fig.3 The representative HPLC-UV chromatogram (λ = 254 nm) of *Catalpa speciosa* bark extract; 1 - gallic acid (tR=3.2 min), 2 - protocatechuic acid (tR=6.3 min), 3 – catechin (tR=8.8 min), 4- p-hydroxybensoic acid (tR=11.8 min), 5 - vanillic acid(tR=12 min), 6 -caffeic acid (tR=15.2 min), 7 - p-coumaric acid(tR=26 min), 8 - ferulic acid(tR=34.4 min).

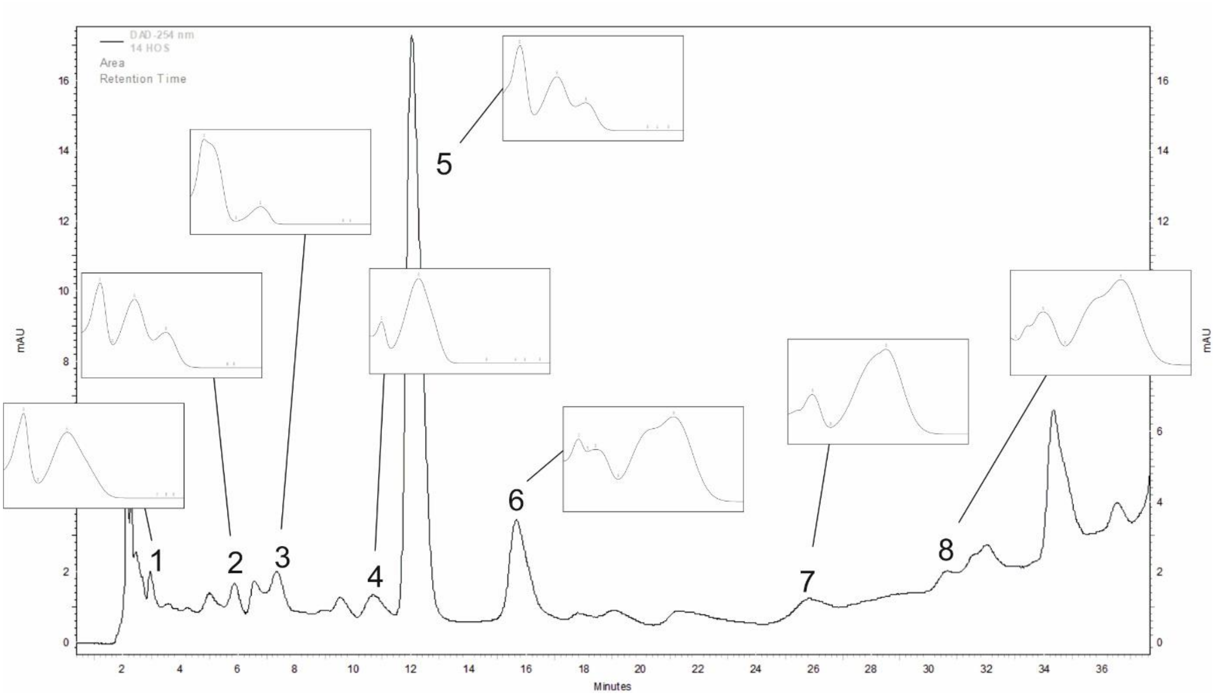

Supplement: Supplementary file 1 [file molecules-24-00412-s001.pdf]
